# Effect of Replacing Animal Protein with Plant Protein on Glycemic Control in Diabetes: A Systematic Review and Meta-Analysis of Randomized Controlled Trials

**DOI:** 10.3390/nu7125509

**Published:** 2015-12-01

**Authors:** Effie Viguiliouk, Sarah E. Stewart, Viranda H. Jayalath, Alena Praneet Ng, Arash Mirrahimi, Russell J. de Souza, Anthony J. Hanley, Richard P. Bazinet, Sonia Blanco Mejia, Lawrence A. Leiter, Robert G. Josse, Cyril W.C. Kendall, David J.A. Jenkins, John L. Sievenpiper

**Affiliations:** 1 Toronto 3D Knowledge Synthesis and Clinical Trials Unit, Clinical Nutrition and Risk Factor Modification Center, St. Michael’s Hospital, Toronto, ON M5C 2T2, Canada; effie.viguiliouk@mail.utoronto.ca (E.V.); sarahe.stewart@mail.utoronto.ca (S.E.S.); viranda.jayalath@mail.utoronto.ca (V.H.J.); alena.ng@mail.utoronto.ca (A.P.N.); smirrahimi@qmed.ca (A.M.); rdesouz@mcmaster.ca (R.J.S.); BlancoMejiaS@smh.ca (S.B.M.); LeiterL@smh.ca (L.A.L.); JosseRG@smh.ca (R.G.J.); cyril.kendall@utoronto.ca (C.W.C.K.); NutritionProject@smh.ca (D.J.A.J.); 2 Department of Nutritional Sciences, Faculty of Medicine, University of Toronto, Toronto, ON M5S 2E8, Canada; anthony.hanley@utoronto.ca (A.J.H.); richard.bazinet@utoronto.ca (R.P.B.); 3 Department of Surgical Oncology, Princess Margaret Cancer Center, University Health Network, Toronto, ON M5G 2C4, Canada; 4 Undergraduate Medical Education, University of Toronto, Toronto, ON M5S 2E8, Canada; 5 School of Medicine, Faculty of Health Sciences, Queen’s University, Kingston, ON K7L 3N6, Canada; 6 Department of Clinical Epidemiology & Biostatistics, Faculty of Health Sciences, McMaster University, Hamilton, ON L8S 4L8, Canada; 7 Leadership Sinai Centre for Diabetes, Mount Sinai Hospital, Toronto, ON M5G 1X5, Canada; 8 Department of Medicine, University of Toronto, Toronto, ON M5S 2E8, Canada; 9 Dalla Lana School of Public Health, University of Toronto, Toronto, ON M5S 2E8, Canada; 10 Division of Endocrinology and Metabolism, St. Michael’s Hospital, Toronto, ON M5C 2T2, Canada; 11 Li Ka Shing Knowledge Institute, St. Michael’s Hospital, Toronto, ON M5C 2T2, Canada; 12 College of Pharmacy and Nutrition, University of Saskatchewan, Saskatoon, SK S7N 5A2, Canada

**Keywords:** plant protein, animal protein, diabetes, glycemic control

## Abstract

Previous research on the effect of replacing sources of animal protein with plant protein on glycemic control has been inconsistent. We therefore conducted a systematic review and meta-analysis of randomized controlled trials (RCTs) to assess the effect of this replacement on glycemic control in individuals with diabetes. We searched MEDLINE, EMBASE, and Cochrane databases through 26 August 2015. We included RCTs ≥ 3-weeks comparing the effect of replacing animal with plant protein on HbA_1c_, fasting glucose (FG), and fasting insulin (FI). Two independent reviewers extracted relevant data, assessed study quality and risk of bias. Data were pooled by the generic inverse variance method and expressed as mean differences (MD) with 95% confidence intervals (CIs). Heterogeneity was assessed (Cochran Q-statistic) and quantified (*I*^2^-statistic). Thirteen RCTs (*n* = 280) met the eligibility criteria. Diets emphasizing a replacement of animal with plant protein at a median level of ~35% of total protein per day significantly lowered HbA_1c_ (MD = −0.15%; 95%-CI: −0.26, −0.05%), FG (MD = −0.53 mmol/L; 95%-CI: −0.92, −0.13 mmol/L) and FI (MD = −10.09 pmol/L; 95%-CI: −17.31, −2.86 pmol/L) compared with control arms. Overall, the results indicate that replacing sources of animal with plant protein leads to modest improvements in glycemic control in individuals with diabetes. Owing to uncertainties in our analyses there is a need for larger, longer, higher quality trials. Trial Registration: ClinicalTrials.gov registration number: NCT02037321.

## 1. Introduction

Diabetes association guidelines (*i.e.*, American Diabetes Association and European Association for the Study of Diabetes) do not currently recommend the intake of major sources of plant protein such as soy, soy-derived foods (e.g., tofu) and nuts for optimal glycemic control [1,2]. One exception is dietary pulses (*i.e.*, beans, peas, chick peas, and lentils), which have recently been recommended by the Canadian Diabetes Association clinical practice guidelines for improving glycemic control in individuals with type 2 diabetes (T2D) [3]. Plant proteins are a major component of vegan and/or vegetarian dietary patterns and have been shown in prospective cohort and cross-sectional studies in Seventh-day Adventists to be associated with lower diabetes risk [4,5] and all-cause mortality [6]. Evidence from a systematic review and meta-analysis of RCTs also suggests that vegetarian diets may improve glycemic control in individuals with T2D [7]. Furthermore, a recent prospective cohort study conducted in over 92,000 women from the Nurses’ Health Study II and over 40,000 men from the Health Professionals Follow-up Study found that substituting 5% energy from plant protein for animal protein was associated with an 18% reduced risk for T2D and substituting 1 serving per day of plant protein foods for animal protein foods was associated with 10%–21% reduced risk for T2D [8]. On the contrary, evidence from previous meta-analyses of prospective cohort studies [9,10], as well as more recent prospective cohort studies [11] have shown that diets higher in animal protein, specifically in red meat, are associated with an increased incidence of T2D. A recent review has even suggested that meat consumption be considered as a risk factor for T2D [12]. However, it is unclear whether the replacement of animal with plant protein would confer glycemic control benefits in individuals with diabetes. Evidence from RCTs remains inconsistent: some trials have shown replacement of animal with plant protein significantly improved glycemic control [13,14], whereas others have shown no effect [15,16]. We therefore conducted a systematic review and meta-analysis of RCTs to synthesize the effect of replacing sources of animal protein with plant protein on glycemic control assessed by HbA_1c_, fasting glucose, and fasting insulin in individuals with diabetes.

## 2. Methods 

We followed the Cochrane Handbook for Systematic Reviews of Interventions for the planning and conduct of this meta-analysis [17]. Reporting followed the Preferred Reporting Items for Systematic Reviews and Meta-Analyses (PRISMA) guidelines [18]. The review protocol is available at ClinicalTrials.gov (registration number: NCT02037321).

### 2.1. Data Sources and Searches

We searched the databases MEDLINE [19], EMBASE [20], and the Cochrane Central Register of Controlled Trials [21] through 26 August 2015 using the search strategy shown in Table S1. Manual searches of reference lists of review articles and included trials supplemented the electronic database searches.

### 2.2. Study Selection

We included RCTs that compared a diet emphasizing the replacement of animal protein sources (e.g., meat, dairy, *etc.*) with major sources of plant protein (e.g., legumes, nuts, *etc.*) on HbA_1c_, fasting glucose, and/or fasting insulin to a control diet without this replacement matched for energy (isocaloric) for a follow-up duration ≥3 weeks in individuals with diabetes (type 1 diabetes (T1D) and/or T2D). Trials that consisted of a non-randomized treatment allocation, <3-weeks follow-up duration, non-isocaloric comparisons, lacked a suitable control (*i.e.*, plant protein source in intervention arm did not replace an animal protein source in the control arm), were not conducted in individuals with diabetes, or did not provide suitable endpoint data were excluded. No restrictions were placed on language.

### 2.3. Data Extraction and Quality Assessment

Two investigators (Effie Viguiliouk and one of: Sarah E. Stewart, Alena Praneet Ng , Viranda H. Jayalath or Arash Mirrahimi) independently reviewed all reports that met the inclusion criteria. A standardized form was used to extract relevant information on trial characteristics and endpoint data. Trial characteristics included: sample size, participant characteristics (e.g., health status, sex, age, *etc.*), study setting (outpatient or inpatient and country), design (crossover or parallel), level of feeding control (metabolically controlled, partially metabolically controlled, or dietary advice), intervention arm (plant protein type), percent and grams per day of animal protein replaced with plant protein (from total protein), control arm (animal protein type), food form (whole food or powder), macronutrient breakdown of background diet(s), energy balance (neutral, positive or negative), follow-up duration, and funding source type (agency, industry, or both). Where available, the mean ± standard deviations (SD) for baseline, end, and change from baseline values, as well as mean differences (MD) were extracted for the primary endpoints (HbA_1c_, fasting glucose, and fasting insulin). Missing SDs were calculated from other available data (95% confidence intervals (CIs), *p*-values, *t* or *F* statistics, standard error (SE)) using standard formulae recommended by the Cochrane Collaboration [17]. Authors were contacted to provide missing data. 

The quality of each trial was assessed using the Heyland Methodological Quality Score (MQS), where a maximum score of 13 points could be received on the basis of information provided for the trials study design, sample and intervention [22]. Trials receiving scores of ≥8 and <8 were considered to be of higher and lower quality, respectively. Disagreements on MQS scores were resolved by consensus. The risk of bias of each trial was assessed using the Cochrane Risk of Bias Tool [17]. Domains of bias assessed were: sequence generation, allocation concealment, blinding, outcome data, and outcome reporting. Trials were marked as “high risk of bias” when the methodological flaw was likely to have affected the true outcome, “low risk of bias” if the flaw was deemed inconsequential to the true outcome, and “unclear risk of bias” when insufficient information was provided to permit judgment. For trials reported exclusively in a published abstract [23] study quality and risk of bias were not assessed. All disagreements were resolved by consensus.

### 2.4. Data Synthesis and Analysis

Data for primary analyses were conducted using Review Manager (RevMan), version 5.3 (The Nordic Cochrane Centre, The Cochrane Collaboration, Copenhagen, Denmark). The difference between the intervention and control arm’s change from baseline value was calculated from each trial for the each of the three primary endpoints. If change from baseline values were not available, end-of-treatment values were used. In the case where we were not able to convert data into appropriate units (*i.e.*, percent change from baseline values), individual patient data was requested [23,24]. Paired analyses were conducted for all crossover trials [25]. If SDs were missing for crossover trials and no other data were available for their derivation, and where we could not derive a calculated pooled correlation coefficient for imputing missing SDs (<5 trials reporting sufficient data) we assumed a correlation coefficient of 0.5, as it is a conservative estimate for an expected range of 0–1. A correlation coefficient of 0.5 was assumed in all three primary analyses due to insufficient data. Once the values were derived from each trial they were pooled and analyzed for each primary endpoint using the generic inverse variance method with random effects models. This approach was used even in the absence of statistically significant between-study heterogeneity, as they yield more conservative summary effect estimates in the presence of residual heterogeneity. Results were expressed as MD with 95% CIs. A two-sided *p*-value < 0.05 was set as the level of significance.

Inter-study heterogeneity was tested using the Cochran Q-statistic and quantified using the *I*^2^-statistic with a significance level set at *p* < 0.10. An *I*^2^ < 50%, *I*^2^ ≥ 50% and *I*^2^ ≥ 75% were considered to be evidence of “moderate”, “substantial” and “considerable” heterogeneity, respectively [17]. Sources of heterogeneity were explored using sensitivity and subgroup analyses. To determine whether a single trial exerted an undue influence on the overall results, sensitivity analyses were performed in which each individual trial was removed from the meta-analysis and the effect size recalculated with the remaining trials. Sensitivity analyses were also undertaken using correlation coefficients of 0.25 and 0.75 to determine whether the overall results were robust to the use of an assumed correlation coefficient of 0.5. *A priori* subgroup analyses (categorical and continuous) were conducted for baseline values of HbA_1c_, fasting glucose, and fasting insulin within the intervention arm, plant protein type, animal protein type, percent and grams of animal protein replaced with plant protein (from total protein), absolute fibre and saturated fat intake within the intervention arm, difference in fibre and saturated fat intake between the intervention and control arm, change from baseline fibre and saturated fat intake within the intervention arm, health status (diabetes type), design, follow-up duration, and study quality (MQS). *Post-hoc* continuous or categorical subgroup analyses were conducted for isoflavone intake, body weight, sex, food form, risk of bias, diabetes duration and diabetes complications. A *post-hoc* analysis consisting of a piecewise linear meta-regression was performed using the *mkspline* function in order to identify a dose-threshold (breakpoints) for the continuous subgroup looking at percent animal protein replaced with plant protein (from total protein) on fasting glucose. 

Publication bias was investigated by visual inspection of funnel plots, quantitative assessment using Egger and Begg tests, and Duval and Tweedie nonparametric “trim-and-fill” analyses, where a *p*-value < 0.05 was considered evidence of small study effects. 

All meta-regressions (*a priori* and *post-hoc*) and publication bias analyses were conducted using STATA software, version 13.0 (StataCorp, College Station, TX, USA) with a significance set at *p* < 0.05.

## 3. Results 

### 3.1. Search Results

**Figure 1:**
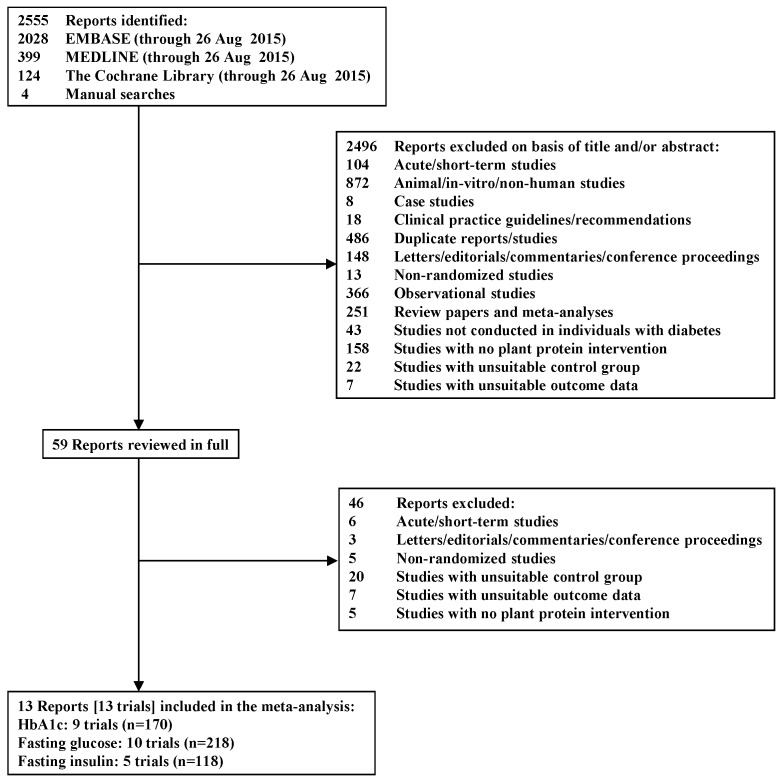
Flow diagram depicting the literature search and selection process.

Figure 1 shows the literature search and selection process. We identified a total of 2555 reports, 2496 of which were determined to be irrelevant based on review of titles and abstracts. The remaining 59 reports were retrieved and reviewed in full, of which 46 were excluded. One of these reports was excluded for an irreconcilable discrepancy in the reporting of the results in two places in the same report [26]. A total of 13 reports containing data from 13 trials in 280 participants with T1D (*n* = 21) [27,28] and T2D (*n* = 256) [13,14,15,16,23,24,29,30,31,32,33] met the eligibility criteria and were included in the analyses. Nine of these trials reported data for HbA_1c_ (*n* = 170) [15,16,23,24,27,28,29,30,32], 10 for fasting glucose (*n* = 218) [13,14,16,24,27,28,30,31,32,33], and five for fasting insulin (*n* = 118) [14,16,24,30,32].

### 3.2. Trial Characteristics 

Table 1 shows the characteristics of the 13 included trials [13,14,15,16,23,24,27,28,29,30,31,32,33]. Trials were conducted in outpatient settings across six countries: Iran (5 trials [13,14,24,31,33]), United States (4 trials [15,28,29,30]), and one trial each from Canada [32], Denmark [16], Germany [23] and Greece [27]. All trials were randomized and majority (77%) used a crossover design [14,15,16,24,27,28,29,31,32,33]. Participants tended to be middle aged (median age: 62 years (range: 30–66 years)), overweight or obese (median BMI: 29 kg/m^2^ (range: 23.8–35.1 kg/m^2^)), with an approximately equal distribution of men and women across trials (median % women: 46% (range: 0%–78% women)). Most trials were conducted in individuals with T2D with the exception of 2 trials conducted in individuals with T1D [27,28]. Approximately half of the trials reported presence of microvascular complications (e.g., nephropathy, retinopathy) among participants [13,15,16,24,29,31]. Median baseline HbA_1c_, fasting glucose, and fasting insulin were 7.2% (range: 5.9%–8.4%), 8.0 mmol/L (range: 6.7–10.4 mmol/L), and 70.2 pmol/L (56.3–134.2 pmol/L), respectively. Mean diabetes duration varied from ~1 to 10 years [13,15,16,29,30,31,32,33] for those with T2D and 15 years [28] or before the onset of 30 years of age [27] for those with T1D; otherwise diabetes duration was unspecified [14,23,24]. The majority of trials did not explicitly provide information on how diabetes was defined with the exception of four trials, in which T2D was defined as fasting plasma glucose ≥7 mmol/L [13,14,30] or the use of oral glucose-lowering agents or insulin [13,14,24,32]. Trials conducted in individuals with T2D had all participants treated with oral glucose-lowering agents (4 trials [15,16,30,33]), some treated with either oral glucose-lowering agents, insulin or both (3 trials [13,24,31]), all treated with insulin (1 trial [29]), or all treated with diet and lifestyle alone (1 trial [32]); otherwise information on the use of oral glucose-lowering agents or insulin was unspecified (2 trials [14,23]). All participants with T1D were treated with insulin (2 trials [27,28]). Five trials required participants to keep their medications stable throughout the trial [14,24,30,31,32], while four trials reported no changes in medication use in most patients [14,15,16,24,28,29,30,31,32]; otherwise it was unspecified [13,23,27,33]. 

Laboratory measurements of glycemic endpoints across trials varied. HbA_1c_ was measured by high-performance liquid chromatography (HPLC) in three trials [16,27,32] and immunoassay in three trials [15,24,30]; otherwise it was unspecified [23,28,29]. Fasting glucose was measured by enzymatic methods across all 10 trials [13,14,16,24,27,28,30,31,32,33]. Fasting insulin was measured by a radioimmunoassay in one trial [30] and an immunoassay in three trials [16,24,32]; otherwise it was unspecified [14]. None of the trials reported using National Glycohemoglobin Standardization Program (NGSP) or International Federation of Clinical Chemistry (IFCC) certified methods.

The majority of trials consisted of a partial replacement of animal with plant protein, with the exception of two trials, which did a full replacement [15,27]. The types of animal protein replaced with plant protein varied among the trials: seven trials (54%) replaced mixed sources of animal protein with mixed sources of plant protein [15,27], soy protein [13,28,29,31] or pulses (*i.e.*, beans, peas, chick peas, and lentils) [23]; four trials (31%) replaced sources of dairy protein with sources of soy protein [16,24,32] or almonds [30]; and two trials (15%) replaced sources of red meat with pulses [14,33]. The animal and plant protein sources were consumed in the form of whole foods in majority of the trials with the exception of three trials [16,23,32], where two of them consisted of animal and plant sources exchanged in the form of isolated protein powders [16], and one of them exchanged in both whole and isolated protein powder forms [23]. The median percentage of animal protein replaced with plant protein from total protein was ~35% per day (range: 4%–70% per day).

The background diets for the intervention and control arms were similar, where mainly the source of protein differed between the two arms. The background diets of the intervention arms consisted of 40%–70% energy (E) from carbohydrate, 9%–30% E from protein, and 20%–40% E from fat with a median fibre and saturated fat intake of 22 g/day (range: 13–41 g/day) and 7.6% E (range: 3%–10% E), respectively. The background diets of the control arms consisted of 40%–69% E from carbohydrate, 9%–30% E from protein, and 22%–37% E from fat with a median fibre and saturated fat intake of 24 g/day (range: 7.7–42 g/day) and 8.4% E (range: 4%–12% E), respectively. In terms of feeding control, one trial was metabolically controlled (*i.e.*, all foods were provided) [15], nine trials were partially metabolically controlled (*i.e.*, test foods/supplements were provided) [13,16,23,28,29,30,31,32], and two trials were not metabolically controlled (*i.e.*, dietary advice was provided) [14,33]; otherwise it was unspecified [27]. The median follow-up duration was eight-weeks (range: four weeks–four years).

The majority of trials (85%) were considered to be of poor quality (MQS < 8). Absence of double-blinding and a preselected or indeterminate sample selection contributed to lower scores (Table S2). Majority of the trials (>75%) were judged as having a “low” or “unclear risk bias” in majority of the domains measured by the Cochrane Risk of Bias Tool. Five trials (42%) were considered “high risk of bias” due to incomplete outcome data [13,14,15,28,31] (Figure S1). In terms of sources of funding, five trials (42%) were funded by agency alone [14,24,30,31,33]; four trials (33%) were funded by both agency and industry [15,16,28,32] and for four trials funding information was unspecified [13,23,27,29].

### 3.3. Hemoglobin A1c (HbA_1c_)

**Figure 2:**
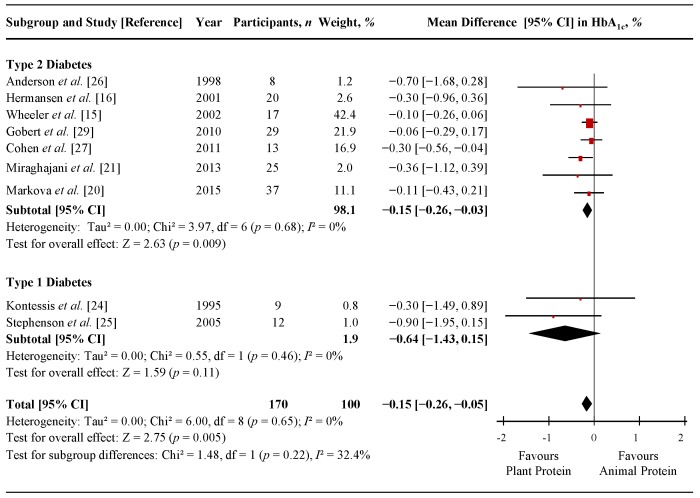
Forest plot of randomized controlled trials investigating the effect of replacing sources of animal with plant protein in individuals with diabetes on hemoglobin A1c (HbA_1c_). Pooled effect estimates for each subgroup and overall effect are represented by the diamonds. Data are expressed as weighted mean differences with 95% confidence intervals (CIs), using the generic inverse-variance method with random effects models. Paired analyses were applied to all crossover trials. Inter-study heterogeneity was tested by the Cochran Q-statistic and quantified by *I*^2^ at a significance level of *p* < 0.10; d*f*, degrees of freedom.

Figure 2 shows a forest plot of the pooled effect of replacing animal with plant protein on HbA_1c_ in individuals with T1D and T2D. Diets emphasizing this replacement significantly lowered HbA_1c_ in comparison to control diets (MD = −0.15% (95% CI: −0.26, −0.05%); *p* = 0.005), with no significant evidence of inter-study heterogeneity (*I*^2^ = 0%; *p* = 0.65). Systematic removal of individual trials did not alter the results. Sensitivity analyses using different correlation coefficients in paired analyses of crossover trials (0.25 and 0.75) did not alter the significance of the pooled effect size.

Table S3 and Figures S2 and S3 show the results of continuous and categorical subgroup analyses for the effect of replacing sources of animal with plant protein on HbA_1c_. Meta-regression analyses did not reveal any statistically significant subgroup effects.

**Table 1:** Characteristics of included randomized controlled trials.

Study, Year (References)	Participants	Age *, Year	Body Weight or BMI *^,†^	Diabetes Duration *, Year	Setting ^‡^	Design	Feeding Control ^§^	PP Type ^||^	AP Type ^||^	Amount of AP Replaced ^¶^	Food Form ^†††^	Diet ^#^	Energy Balance	Follow-up	MQS **	Funding Sources ††
**TYPE 2 DIABETES**																
**Anderson *et al.* 1998 [29] ^‡‡^**	8 T2D+N, O, HT (M only)	68 (18.4)	111 (66.8) kg	>5	OP, USA	C	Supp			50% (~55.5 g/day)	Whole		Neutral	8 wk	8	NA
Intervention								Soy				~15:55:30				
Control									Mixed			~15:55:30				
**Hermansen *et al.* 2001 [16] ^§§^**	20 T2D+R (6W, 14M)	63.6 (7.5)		3 (2.7)	OP, DNK	C	Supp			~33% (50 g/day)	Powder		Neutral	6 wk	5	Agency-industry
Intervention			88.7 (11.9) kg					Soy				25:41:29				
Control			88.3 (11.8) kg						Casein			26:43:28				
**Wheeler *et al.* 2002 [15] ^||||^**	17 T2D+N (3W, 14M)	56 (12.4)	102.3 (21.4) kg	7 (1–16)	OP, USA	C	Met			60% (64 g/day)	Whole		Neutral	6 wk	6	Agency-industry
Intervention								Mixed				17:53:30				
Control									Mixed			17:53:30				
**Azadbakht *et al.* 2008 [13] ^¶¶^**	41 T2D+N,R (23W, 18M)			10 (3)	OP, IRN	P	Supp			35% (~20 g/day)	Whole		Neutral	4 y	4	NA
Intervention		61.9 (11.8)	71 (9) kg					Soy				~10:70:20				
Control		62.1 (12.1)	72 (8) kg						Mixed			~10:68:22				
**Azadbakht *et al.* 2009 [31] ##**	14 T2D+N (4W, 10M)	62.5 (12.1)		10 (4)	OP, IRN	C	Supp			35% (~20 g/day)	Whole		Neutral	7 wk	4	Agency
Intervention			70.6 (10.3) kg					Soy				~9:70:21				
Control			70.7 (10.7) kg						Mixed			~9:69:22				
Gobert *et al.* 2010 [32] ***	29 T2D (13W, 16M)	60.1 (9.64)	83.4 (10.9) kg	3.4 (4.8)	OP, CAN	C	Supp			~34% (40 g/day)	Powder		Neutral	8 wk	7	Agency-industry
Intervention								Soy				~23:45:32				
Control									Milk			~23:44:33				
**Cohen *et al.* 2011 [30] ^‡‡‡^**	13 T2D (6W, 7M)			At least 1 year	OP, USA	P	Supp			NA (~6 g/day)	Whole	NA	Neutral	12 wk	7	Agency
Intervention		66 (8.1)	96.1 (21.8) kg					Almonds								
Control		66 (8.7)	105.1 (29.6) kg						Cheese							
**Miraghajani *et al.* 2013 [24] ^§§§^**	25 T2D+N (15W, 10M)	51 (10)		NA	OP, IRN	C	DA			~4% (2.5 g/day)	Whole		Neutral	4 wk	6	Agency
Intervention			76.1 (13.2) kg					Soy				14:46:40				
Control			76.5 (13.6) kg						Milk			13:50:37				
**Abd-Mishani *et al.* 2014 [33] ^|||||||^**	21 T2D (18W, 6M)	61.7 (6)	74.5 (7.1) kg	3.4 (1.2)	OP, IRN	C	DA			~18% (~13.3 g/day)	Whole		Neutral	8 wk	4	Agency
Intervention								Pulses				15:55:30				
Control									Red meat			15:55:30				
**Hosseinpour-Niazi *et al.* 2014 [14] ^|||||||^**	31 T2D+O (24W, 7M)	58.1 (33.4)		NA	OP, IRN	C	DA			~17% (~13.3 g/day)	Whole		Neutral	8 wk	6	Agency
Intervention			27.7 (3.34) kg/m^2^					Pulses				14:54:33				
Control			27.8 (3.34) kg/m^2^						Red meat			15:52:34				
**Markova *et al.* 2015 [23] ^¶¶¶^**	37 T2D (13W, 24M)			NA	OP, DEU	P	Supp			~32.5% (NA)	Both		Neutral	6 wk	NA	NA
Intervention		63.7 (6.54)	86 (13.9) kg					Pulses				30:40:30				
Control		65 (5.9)	92.6 (11.9) kg						Mixed			30:40:30				
**TYPE 1 DIABETES**																
**Kontessis *et al.* 1995 [27] ^###^**	9 T1D (7W, 2M)	32 (20–48) ****	23.8 (20.6–27.8) kg/m^2^ ****	Onset before the age of 30	OP, GRC	C	NA			70% (~49 g/day)	Whole		Neutral	4 wk	7	NA
Intervention								Mixed				~17:49:34				
Control									Mixed			~19:41:37				
**Stephenson *et al*, 2005 [28]**	12 T1D+GHF (6W, 6M)	29.9 (8)	79.0 (5.9) kg	15.1 (8)	OP, USA	C	Supp			~46%–56% (45–55 g/day)	Whole		Neutral	8 wk	5	Agency-industry
Intervention								Soy ^††††^				22:53:27				
Control									Mixed			16:49:36				

AP = animal protein; BW = body weight; C = crossover; CAN = Canada; DA = dietary advice; DEU = Germany; DNK = Denmark; GHF = glomerular hyperfiltration; GRC = Greece; HT = hypertension; IRN = Iran; M = men; Met = metabolic feeding control; MQS = Heyland Methodological Quality Score; N = nephropathy; NA = data not available; O = overweight and/or obese; P = parallel; PP = plant protein; R = retinopathy; Supp = supplemental feeding control; T1D = type 1 diabetes; T2D = type 2 diabetes; USA = United States of America; W = women; wk = weeks; y = years; * Age, body weight or BMI, and diabetes duration are reported as mean ± SD or range; ^†^ Baseline body weight (kg) values. Baseline BMI values (kg/m^2^) are only reported when no data on body weight were available; ^‡^ Countries are abbreviated using three letter country codes (ISO 3166-1 alpha-3 codes); ^§^ Metabolic feeding control (Met) is the provision of all meals, snacks, and study supplements consumed during the study under controlled conditions. Supplement feeding control (Supp) is the provision of study supplements only. Dietary advice (DA) is the provision of counselling on the appropriate test and control diets; ^||^ Plant and animal protein types refer to the specific sources of plant proteins prescribed by the study to replace a specific source(s) of animal protein. If the prescribed plant or animal protein type was not specified by the study it was assumed that the protein type consisted of mixed sources. For example, Kontessis *et al.* [27] referred to their intervention arm as a “vegetable protein diet” and their control arm as an “animal protein diet”; therefore it was assumed that the intervention and control arm consisted of mixed sources of plant and animal protein, respectively; ^¶^ Data in this column represents the amount of plant protein that was prescribed by the study to replace animal protein. Numbers not in parentheses represent the percentage of total protein replacing animal protein with plant protein. Numbers in parentheses represent the amount of plant protein prescribed/amount of animal protein replaced in grams per day. Numbers preceded by “~” were calculated using relevant data provided by the study. All studies partially replaced animal protein with plant protein with the exception of Wheeler *et al.* [15] and Kontessis *et al.* [27], which fully replaced animal protein with plant protein sources; ^#^ Data in this column indicates the designed percent energy breakdown from protein:carbohydrate:fat reported from each study. If these values were not available or provided, the measured percent energy breakdown from the end of the study were provided. Numbers preceded by “~” were calculated using relevant data provided by the study; ** Trials with a MQS score ≥ 8 were considered to be of higher quality; ^††^ Agency funding consists of funding from government, university, or not-for-profit health agency sources. None of the trialists declared any conflicts of interest with the exception of Stephenson *et al.* [28] and Hermansen *et al.* [16]; ^‡‡^ Both intervention and control arm were designed to contain 1 gram of protein per kilogram body weight per day. Fifty percent of the protein in the soy protein intervention arm was in the form of beverages, meat analogue patties, or ground meat analogues, whereas 50% of the protein in the animal protein control arm was in the form of ground beef with a specified fat content and cow milk; ^§§^ Both intervention and control arm were provided with their respective powders and instructed to mix half of their daily allotted amount in 250ml of water before breakfast and half before their evening meal. The powder provided in the intervention arm also contained 20 g/day of soy cotyledon fibre and a high isoflavone content (minimum 165 mg/day), whereas the powder provided in the control arm contained 20 g/day of cellulose; ^||||^ The intervention arm consisted exclusively of plant protein (62% soy-based), where major protein foods included tofu, textured vegetable protein, soy milk, and legumes. The control arm consisted of 60% animal protein and 40% plant protein, where major protein foods included beef, poultry, fish and milk; ^¶¶^ 73% of participants in this study had retinopathy. Both intervention and control arm were designed to contain 0.8 grams of protein per kilogram body weight per day. The intervention arm consisted of 35% soy protein (in the form of textured soy protein), 30% other plant protein and 35% animal protein, whereas the control arm consisted of 70% animal protein and 30% plant protein; ^##^ The intervention arm consisted of 35% soy protein (in the form of textured soy protein), 30% other plant protein and 35% animal protein and the control arm consisted of 70% animal protein and 30% plant protein;*** Women in this study were postmenopausal. The powder provided in the intervention arm also consisted of 88 mg/day isoflavones (65% genistein, 31% daidzein, 4% glycitein). Participants supplemented their habitual diets with the powders and were provided with multiple examples of ways to consume them but were encouraged to reconstitute them with water with the option of adding Nestle flavour packets. In order to avoid excess protein and calcium intakes, participants were counselled to replace foods like milk, cheese, and lunchmeat with the powders; ^†††^ Food form refers to whether the test foods in each study were in the form of whole foods (whole), isolated protein powders (powder), or both; ^‡‡‡^ Participants were instructed to consume their food prescription (1 oz almonds or 2 cheese sticks) 5 days per week; ^§§§^ Both intervention and control arm were designed to contain 0.8 grams of protein per kilogram body weight per day. The intervention and control arm consisted of 1 glass of soy and cow’s milk (240 mL each) per day, respectively; ^|||||||^ Both intervention and control arm were prescribed a Therapeutic Lifestyle Change (TLC) diet. The intervention arm was the same as the control arm but participants were advised to replace 2 servings of red meat with different types of cooked legumes such as lentils, chickpeas, peas and beans 3 days per week. Half a cup of cooked legumes was considered to be one serving of red meat; ^¶¶¶^ The intervention arm received foods enriched with pea protein (*i.e.*, mash powder, bread, pancake powder, noodles). The control arm contained dairy products and meat in larger quantities to achieve 30% of energy from protein; ^###^ Both intervention and control arm were designed to contain 1 gram of protein per kilogram body weight per day. Intervention arm consisted of plant protein exclusively and the control arm consisted of ~70% animal protein and 30% plant protein. The intervention arm was also provided animal fat supplements, as well as calcium and phosphate tablets; **** Reported as a median value; ^††††^ Nine daily soy food intake options were provided: soy patties; soy pasta; soy chocolate beverage; chocolate, vanilla, or plain silk soy milk; lemon or chocolate soy bars; roasted soy nuts, or frozen edamame.

### 3.4. Fasting Glucose

**Figure 3:**
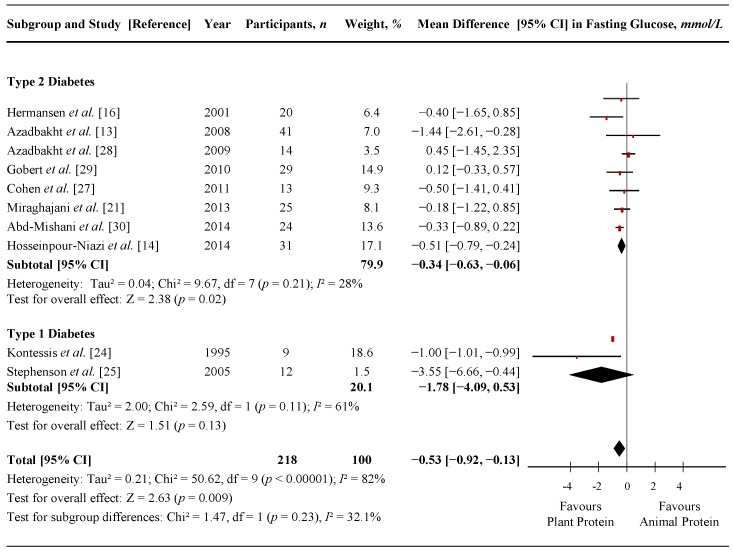
Forest plot of randomized controlled trials investigating the effect of replacing sources of animal with plant protein in individuals with diabetes on fasting glucose. Pooled effect estimates for each subgroup and overall effect are represented by the diamonds. Data are expressed as weighted mean differences with 95% confidence intervals (CIs), using the generic inverse-variance method with random effects models. Paired analyses were applied to all crossover trials. Inter-study heterogeneity was tested by the Cochran Q-statistic and quantified by *I*^2^ at a significance level of *p* < 0.10; d*f*, degrees of freedom.

Figure 3 shows a forest plot of the pooled effect of replacing animal with plant protein on fasting glucose in individuals with T1D and T2D. Diets emphasizing this replacement significantly lowered fasting glucose in comparison to control diets (MD = −0.53 mmol/L (95% CI: −0.92, −0.13 mmol/L); *p* = 0.009) with considerable amount of inter-study heterogeneity (*I*^2^ = 82%; *p* < 0.00001). Systematic removal of individual trials did not alter the results. Sensitivity analyses using different correlation coefficients in paired analyses of crossover trials (0.25 and 0.75) did not alter the significance of the pooled effect size.

Table S4, Table S6 and Figures S4 and S5 show the results of continuous and categorical subgroup analyses for the effect of replacing animal with plant protein on fasting glucose. Three significant subgroups were identified in the categorical subgroup analyses (Figure S4). Evidence of effect modification was seen for animal protein type, where the fasting glucose reduction achieved by replacing mixed animal protein sources with plant protein was significantly greater than the fasting glucose reduction achieved by replacing dairy or red meat protein sources with plant protein (*p* = 0.003). The second significant subgroup showed an effect modification by percent animal protein replaced with plant protein (from total protein), where the fasting glucose reduction in trials that replaced ≥35% of animal protein with plant protein was significantly greater than the fasting glucose reduction when replacing <35% (*p* = 0.025). The third significant subgroup showed an effect modification for diabetes duration, where the fasting glucose reduction in trials conducted in participants with ≥5 years diabetes duration was significantly greater than the fasting glucose reduction in those with <5 years (*p* = 0.006). No other subgroup analyses revealed statistically significant subgroup effects. *Post-hoc* analyses for the continuous subgroup looking at percent animal protein replaced with plant protein (from total protein) on fasting glucose using a piecewise linear meta-regression did not indicate a dose-threshold (Table S6).

### 3.5. Fasting Insulin

**Figure 4:**
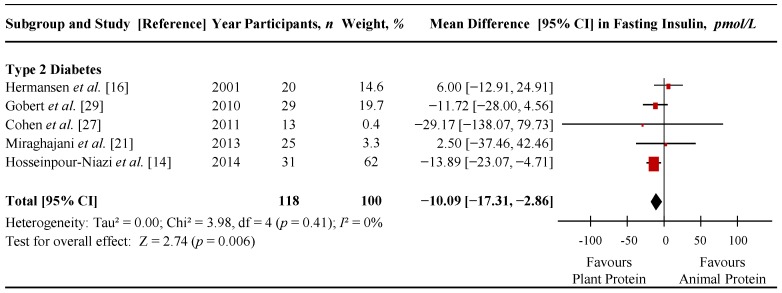
Forest plot of randomized controlled trials investigating the effect of replacing sources of animal with plant protein in individuals with diabetes on fasting insulin. Pooled effect estimates for each subgroup and overall effect are represented by the diamonds. Data are expressed as weighted mean differences with 95% confidence intervals (CIs), using the generic inverse-variance method with random effects models. Paired analyses were applied to all crossover trials. Inter-study heterogeneity was tested by the Cochran Q-statistic and quantified by *I*^2^ at a significance level of *p* < 0.10; d*f*, degrees of freedom.

Figure 4 shows a forest plot of the pooled effect of replacing animal with plant protein on fasting insulin in individuals with T2D. Diets emphasizing this replacement significantly lowered fasting insulin in comparison to control diets (MD = −10.09 pmol/L (95% CI: −17.31, −2.86 pmol/L); *p* = 0.006) with no significant evidence of inter-study heterogeneity (*I*^2^ = 0%; *p* = 0.41). Sensitivity analyses showed that removal of any one of the following 3 trials nullified the significance of the effect estimate: Gobert *et al.* [32] (MD = −6.89 pmol/L (95% CI: −19.20, 5.42 pmol/L), *p* = 0.27; *I*^2^ = 24%, *p* = 0.27); Cohen *et al.* [30] (MD = −8.68 pmol/L (95% CI: −17.90, 0.53 pmol/L), *p* = 0.06; *I*^2^ = 22%, *p* = 0.28) and Hosseinpour-Niazi *et al.* [14] (MD = −3.89 pmol/L (95% CI: −15.61, 7.83 pmol/L), *p* = 0.52; *I*^2^ = 0%, *p* = 0.52). None of these trials included insulin-treated participants. Sensitivity analyses using different correlation coefficients of crossover trials showed that a correlation coefficient of 0.25 did not alter the significance of the pooled effect size, but a correlation coefficient of 0.75 changed the pooled effect size from significant to non-significant (MD = −7.14 pmol/L (95% CI: −16.69, 2.40 pmol/L), *p* = 0.14; *I*^2^ = 38%, *p* = 0.17).

Table S5 and Figures S6 and S7 show the results of continuous and categorical subgroup analyses for the effect of replacing animal with plant protein on fasting insulin. Meta-regression analyses did not reveal any statistically significant subgroup effects.

### 3.6. Publication Bias

**Figure 5:**
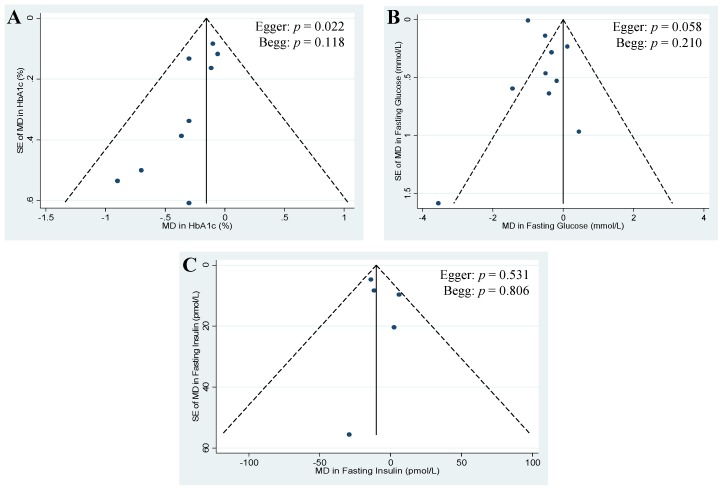
Publication bias funnel plots for HbA_1c_ (**A**); fasting glucose (**B**); and fasting insulin (**C**). The solid line represents the pooled effect estimate expressed as the weighted mean difference (MD) for each analysis; the dashed lines represent pseudo-95% confidence limits; and the circles represent effect estimates for each included study. *p*-values displayed in the top right corner of each funnel plot are derived from quantitative assessment of publication bias by Egger and Begg tests set at a significance level of *p* < 0.05; SE, standard error.

Figure 5A–C shows the funnel plots for each primary endpoint. Visual inspection of funnel plots revealed asymmetry for HbA_1c_ and fasting glucose, suggesting study effects favouring the replacement of animal with plant protein. Egger tests revealed evidence of significant publication bias for HbA_1c_ (*p* = 0.022) and approached significance for fasting glucose (*p* = 0.058). Begg tests did not reveal evidence of significant publication bias for any of the endpoints. With one exception, these tests should be interpreted with caution, as most of them were based on <10 trials [17]. Trim-and-fill analyses for HbA_1c_ and fasting insulin did not identify any potentially missed studies due to publication bias (Figures S8 and S10), however, asymmetry in the funnel plot for fasting glucose was identified, and 1 additional study was “filled” in to mitigate publication bias. With the inclusion of the “filled” study, the MD for fasting glucose was not significantly altered (MD = −0.56 mmol/L (95% CI: −0.94, −0.18 mmol/L); *p* = 0.004; Figure S9).

## 4. Discussion

To the best of our knowledge, this is the first systematic review and meta-analysis of RCTs to assess the effect of replacing sources of animal protein with major sources of plant protein on HbA_1c_, fasting glucose, and fasting insulin in individuals with T1D and T2D. We included 13 RCTs looking at this replacement on these three endpoints in 280 predominantly middle-aged adults. Pooled analyses showed a significant lowering of HbA_1c_ of −0.15%, fasting glucose of −0.53 mmol/L and fasting insulin of −10.09 pmol/L in diets that replaced animal with plant protein at a median level of ~35% of total protein per day over a median follow-up duration of approximately eight weeks.

### 4.1. Findings in Relation to Other Studies

There have been several systematic reviews and meta-analyses of RCTs looking at the effect of specific sources of plant protein (*i.e.*, soy products, dietary pulses, and tree nuts) on glycemic control. In terms of soy products, two meta-analyses have been conducted, one in individuals with T2D [34] and one in individuals with and without diabetes (e.g., healthy, hypercholesterolemia, metabolic syndrome, *etc.*) [35]. Both found no overall significant effect on various glycemic endpoints, however, the direction of the effect favoured soy interventions [34,35]. Two meta-analyses looking at the effect of tree nuts have also been conducted, one in individuals with T2D [36] and one in individuals with and without diabetes [37]. Both found significant improvements in fasting glucose, as well as HbA_1c_ in individuals with T2D [36]. Lastly, there have been a series of meta-analyses conducted looking at the effect of incorporating dietary pulses into the diet alone or in the context of a low-GI or high-fibre diet in individuals with and without diabetes [38]. Pulses alone were found to significantly lower fasting glucose and fasting insulin. In the context of a low-GI and high-fibre diet, pulses were found to significantly lower glycosylated blood proteins (measured as HbA_1c_ or fructosamine), and fasting glucose in the context of a high-fibre diet [38]. Overall, the results of these meta-analyses appear to be consistent with our findings.

### 4.2. Possible Mechanisms of Action

Several potential mechanisms may explain the beneficial effects of replacing animal with plant protein on glycemic control. The reduction in body iron stores may be one such mechanism. As a pro-oxidant, iron catalyzes several cellular reactions that result in the production of reactive oxygen species, which increases oxidative stress and tissue damage, including damage to the pancreatic β-cells [39,40]. Prospective cohort studies have shown that increased heme iron intake, found only in animal protein sources [41], is significantly associated with an increased risk of T2D [42,43], whereas non-heme iron intake, which is found in both plant and animal source foods [41], has been shown to be either inversely associated with [43] or not associated with [42] T2D incidence. This may be attributed to differences in bioavailability between non-heme and heme iron, as heme iron is associated with higher body iron stores [44,45]. Observational studies have shown that serum ferritin, the storage form of iron, predicted the development of hyperglycaemia [46,47] and T2D [46,48] and was found to be positively associated with insulin resistance [46,49,50]. In addition, randomized trials have shown that the use of phlebotomy to reduce serum ferritin levels was associated with improved glucose tolerance in individuals with metabolic syndrome [51] and T2D [52]. Another mechanism may relate to differences in amino acid profiles of animal and plant protein. Compared to animal proteins, plant proteins appear to be higher in l-arginine. Randomized trials have shown that long-term oral administration of l-arginine improves insulin sensitivity in individuals with T2D [53,54] and *in vitro* studies suggest that l-arginine promotes insulin secretion from pancreatic β-cells by stimulating electrical activity [55,56,57,58,59]. Although our findings show improvements for fasting insulin, specific measures of insulin sensitivity were not explored in the present meta-analysis, where only one of the included trials looked at insulin resistance and showed non-significant reductions in HOMA-IR [32]. Neither endpoint, however, is considered to be a good marker of peripheral insulin sensitivity [60] and thus further studies are warranted. Other potential mechanisms may involve a reduction in the glycemic index [61,62], as well as the level of sodium and nitrites found in processed meats [63,64]. 

### 4.3. *A-Priori* and *Post-Hoc* Subgroup Analyses

Evidence of considerable heterogeneity was found in the primary pooled analysis for fasting glucose. Categorical subgroup analyses showed evidence of effect modification by animal protein type (*p* = 0.003), percent animal protein replaced with plant protein (from total protein) (*p* = 0.025), and diabetes duration (*p* = 0.006). Based on the residual *I*^2^ for these three subgroups (98.26%, 35.82%, and 28.04%, respectively), it appears that a large portion of the heterogeneity is explained by the latter two subgroups, which show that trials replacing ≥35% of animal with plant protein and with participants ≥5 years diabetes duration had greater reductions in the mean difference for fasting glucose in comparison to those replacing <35% and with <5 years diabetes duration. Significant effect modification by these two subgroups was not seen in our continuous subgroup analyses, supporting a non-linear relationship. In addition, *post-hoc* analyses on the subgroup looking at percent animal protein replaced with plant protein (from total protein) did not identify a dose-threshold. With regards to the primary pooled analysis for fasting insulin, sensitivity analyses suggest that the primary pooled effect size is not robust when subject to removing individual trials from the pooled analysis or when using a different correlation coefficient.

### 4.4. Limitations

Several limitations exist in the present meta-analysis. First, the majority of trials had small sample sizes. Second, it is uncertain whether the follow-up duration of included trials was sufficient to observe meaningful changes in glycemic control: while HbA_1c_ levels reflect blood glucose control in the preceding three months (∼90 days or 12 weeks) [65], the majority of trials (85%) were shorter than 12-weeks in duration. There was, however, no effect modification by follow-up duration in the subgroup analyses. Third, most trials (85%) were of poor study quality (MQS < 8). There was, however, no effect modification by study quality in subgroup analyses. Fourth, most subgroup analyses were underpowered due to the limited number of available studies. As a result, we cannot rule out important subgroup effects. Fifth, only a small number of trials (15%) focused on glycemic control endpoints as a primary outcome. Sixth, sensitivity analyses showed that the pooled effect estimate for fasting insulin was not robust to the removal of individual trials. Seventh, there was a limited amount of data for individuals with T1D, where only two of the available trials included participants with T1D.

### 4.5. Implications

The reductions seen in HbA_1c_ meets half the threshold proposed by the U.S. Food and Drug Administration for the development of new drugs for diabetes (≥0.3%) [66] and therefore may or may not be clinically significant. It is important to note, however, that the glycemic benefits seen in our meta-analysis are in addition to the use of oral glucose-lowering agents by majority of individuals. Therefore, replacing animal protein with major sources of plant protein may be one strategy that can be combined with standard therapy to help improve and manage glycemic control in individuals with diabetes. Furthermore, the majority of RCTs in our meta-analysis used soy and soy-derived products to replace sources of animal protein, a food that is consumed by only 3.3% of Canadians on any given day, with daily intakes ranging from 1.5 g/day among low consumers to 16.5 g/day among high consumers (<1 serving) [67]. In general, this is consistent with the overall consumption of major plant protein sources in the North American population, which is very low relative to other sources of plant protein, such as grains [68,69,70,71]. Therefore, there is ample room in the diet to increase the intake of other sources of plant protein, such as those from dietary pulses (beans, peas, chickpeas, and lentils).

## 5. Conclusions 

In conclusion, the present systematic review and meta-analysis of RCTs found significant modest improvements in HbA_1c_, fasting glucose and fasting insulin in individuals with diabetes when using major sources of plant protein to replace sources of animal protein at a level ≥35% of total protein per day over a median duration of approximately eight weeks. In order to address the sources of uncertainty and the limitations in our analyses, there is a need for larger, longer, and higher quality RCTs that assess the effect of other sources of plant protein, in addition to soy, in replacement for animal protein at a level ≥35% of total protein on glycemic endpoints as the primary outcome. The inclusion of such RCTs in future meta-analyses will help guide the design of future RCTs in this area, as well as the development of nutrition recommendations and health claims.

## Supplementary Materials

Supplementary materials can be accessed at: http://www.mdpi.com/2072-6643/7/12/5509/s1.
