# Supplementary material for: Effect of Replacing Animal Protein with Plant Protein on Glycemic Control in Diabetes: A Systematic Review and Meta-Analysis of Randomized Controlled Trials
Source: Nutrients. 2015 Dec 1;7(12):9804–24. doi: 10.3390/nu7125509 (PMC4690061; doi:10.3390/nu7125509)
Supplement: Supplementary file 1 [file nutrients-07-05509-s001.docx]

Supplementary Materials: Effect of Replacing Animal Protein with Plant Protein on Glycemic Control in Diabetes: A Systematic Review and Meta-Analysis of Randomized Controlled Trials

Effie Viguiliouk, Sarah E. Stewart, Viranda H. Jayalath, Alena Praneet Ng,
Arash Mirrahimi, Russell J. de Souza, Anthony J. Hanley, Richard P. Bazinet,
Sonia Blanco Mejia, Lawrence A. Leiter, Robert Josse, Cyril W. C. Kendall,
David J. A. Jenkins and John L. Sievenpiper

1. Supplementary Tables

**Table S1.** Search strategy *.

| **Database** | **Search Period** | **Search Terms** |
| --- | --- | --- |
| MEDLINE | 1946 to August 2015 | 1. exp Diet, Vegetarian/  2. vegetarian*.mp.  3. vegan*.mp.  4. exp Vegetable Proteins/  5. (vegetable* adj1 protein*).mp.  6. (plant* adj1 protein*).mp.  7. (plant* adj1 food*).mp.  8. (plant* adj1 based).mp.  9. exp Fabaceae/  10. exp Soybean Proteins/  11. soy*.mp.  12. tofu*.mp.  13. natto*.mp.  14. tempeh*.mp.  15. miso*.mp.  16. lentil*.mp.  17. bean*.mp.  18. legume*.mp.  19. peanut*.mp.  20. (meat* adj1 analog*).mp.  21. lactoovo*.mp.  22. lacto-ovo*.mp.  23. ovolacto*.mp.  24. ovo-lacto*.mp.  25. lactoveg*.mp.  26. lacto-veg*.mp.  27. ovoveg*.mp.  28. ovo-veg*.mp.  29. 1 or 2 or 3 or 4 or 5 or 6 or 7 or 8 or 9 or 10 or 11 or 12 or 13 or 14 or 15 or 16 or 17 or 18 or 20 or 21 or 22 or 23 or 24 or 25 or 26 or 27 or 28  30. omnivor*.mp.  31. (conventional adj3 diet*).mp.  32. (normal adj3 diet*).mp.  33. (regular adj3 diet*).mp.  34. (mixed adj3 diet*).mp.  35. exp Meat/  36. exp Eggs/  37. exp Egg Proteins, Dietary/  38. exp Dairy Products/  39. exp Milk/  40. exp Milk Proteins/  41. (meat* adj1 protein*).mp.  42. (meat* adj1 product*).mp.  43. (animal* adj1 protein*).mp.  44. (animal* adj1 product*).mp.  45. (fish* adj1 protein*).mp.  46. (fish* adj1 product*).mp.  47. (poultry adj1 protein*).mp.  48. (poultry adj1 product*).mp.  49. (chicken* adj1 protein*).mp.  50. (chicken* adj1 product*).mp.  51. (egg* adj1 protein*).mp.  52. (egg* adj1 product*).mp.  53. (milk adj1 protein*).mp.  54. (milk adj1 product*).mp.  55. (dairy adj1 protein*).mp.  56. (dairy adj1 product*).mp.  57. 30 or 31 or 32 or 33 or 34 or 35 or 36 or 37 or 38 or 39 or 40 or 41 or 42 or 43 or 44 or 45 or 46 or 47 or 48 or 49 or 50 or 51 or 52 or 53 or 54 or 55 or 56  58. OGTT.mp.  59. exp Hemoglobin A, Glycosylated/  60. hba1c.mp.  61. fructosamine*.mp.  62. insulin*.mp.  63. glycemia.mp.  64. exp Glucose/  65. exp Hyperglycemia/  66. hyperinsulin*.mp.  67. dysglycemia.mp.  68. gly* albumin.mp.  69. exp Diabetes Mellitus/  70. metabolic syndrome.mp.  71. HOMA*.mp.  72. 58 or 59 or 60 or 61 or 62 or 63 or 64 or 65 or 66 or 67 or 68 or 69 or 70 or 71  73. 29 and 57 and 72  74. limit 73 to animals  75. limit 74 to human  76. 74 not 75  77. 73 not 76  78. 77 not (exp infant formula/ or exp milk, human/) |
| EMBASE | 1947 to August 2015 | 1. exp vegetarian diet/  2. exp vegetarian/  3. vegetarian*.mp.  4. vegan*.mp.  5. exp vegetable protein/  6. (vegetable* adj1 protein*).mp.  7. (plant* adj1 protein*).mp.  8. (plant* adj1 food*).mp.  9. (plant* adj1 based).mp.  10. exp Fabaceae/  11. soy*.mp.  12. tofu*.mp.  13. natto*.mp.  14. tempeh*.mp.  15. miso*.mp.  16. lentil*.mp.  17. bean*.mp.  18. legume*.mp.  19. peanut*.mp.  20. (meat* adj1 analog*).mp.  21. lactoovo*.mp.  22. lacto-ovo*.mp.  23. ovolacto*.mp.  24. ovo-lacto*.mp.  25. lactoveg*.mp.  26. lacto-veg*.mp.  27. ovoveg*.mp.  28. ovo-veg*.mp.  29. 1 or 2 or 3 or 4 or 5 or 6 or 7 or 8 or 9 or 10 or 11 or 12 or 13 or 14 or 15 or 16 or 17 or 18 or 20 or 21 or 22 or 23 or 24 or 25 or 26 or 27 or 28  30. exp omnivore/  31. omnivor*.mp.  32. (conventional adj3 diet*).mp.  33. (normal adj3 diet*).mp.  34. (regular adj3 diet*).mp.  35. (mixed adj3 diet*).mp.  36. exp Meat/  37. exp egg/  38. exp dairy product/  39. (meat* adj1 protein*).mp.  40. (meat* adj1 product*).mp.  41. (animal* adj1 protein*).mp.  42. (animal* adj1 product*).mp.  43. (fish* adj1 protein*).mp.  44. (fish* adj1 product*).mp.  45. (poultry adj1 protein*).mp.  46. (poultry adj1 product*).mp.  47. (chicken* adj1 protein*).mp.  48. (chicken* adj1 product*).mp.  49. (egg* adj1 protein*).mp.  50. (egg* adj1 product*).mp.  51. (milk adj1 protein*).mp.  52. (milk adj1 product*).mp.  53. (dairy adj1 protein*).mp.  54. (dairy adj1 product*).mp.  55. 30 or 31 or 32 or 33 or 34 or 35 or 36 or 37 or 38 or 39 or 40 or 41 or 42 or 43 or 44 or 45 or 46 or 47 or 48 or 49 or 50 or 51 or 52 or 53 or 54  56. exp oral glucose tolerance test/  57. OGTT.mp.  58. exp hemoglobin A1c/  59. hba1c.mp.  60. fructosamine*.mp.  61. insulin*.mp.  62. exp glucose blood level/  63. glycemia.mp.  64. exp glucose/  65. 'impaired fasting glucose'.mp.  66. hyperglycemia.mp.  67. 'impaired glucose tolerance'.mp.  68. hyperinsulin*.mp.  69. dysglycemia.mp.  70. 'gly* albumin'.mp.  71. exp diabetes mellitus/  72. exp insulin dependent diabetes mellitus/  73. exp non insulin dependent diabetes mellitus/  74. exp pregnancy diabetes mellitus/  75. exp metabolic syndrome X/  76. HOMA*.mp.  77. 56 or 57 or 58 or 59 or 60 or 61 or 62 or 63 or 64 or 65 or 66 or 67 or 68 or 69 or 70 or 71 or 72 or 73 or 74 or 75 or 76  78. 29 and 55 and 77  79. limit 78 to (animals and animal studies)  80. 78 not 79  81. 80 not (exp breast milk/ or exp infant formula/) |
| Cochrane Central Register of Controlled Trials | through to 26 August 2015 | 1. exp Diet, Vegetarian/  2. vegetarian*.mp.  3. vegan*.mp.  4. exp Vegetable Proteins/  5. (vegetable* adj1 protein*).mp.  6. (plant* adj1 protein*).mp.  7. (plant* adj1 food*).mp.  8. (plant* adj1 based).mp.  9. exp Fabaceae/  10. exp Soybean Proteins/  11. soy*.mp.  12. tofu*.mp.  13. natto*.mp.  14. tempeh*.mp.  15. miso*.mp.  16. lentil*.mp.  17. bean*.mp.  18. legume*.mp.  19. peanut*.mp.  20. (meat* adj1 analog*).mp.  21. lactoovo*.mp.  22. lacto-ovo*.mp.  23. ovolacto*.mp.  24. ovo-lacto*.mp.  25. lactoveg*.mp.  26. lacto-veg*.mp.  27. ovoveg*.mp.  28. ovo-veg*.mp.  29. 1 or 2 or 3 or 4 or 5 or 6 or 7 or 8 or 9 or 10 or 11 or 12 or 13 or 14 or 15 or 16 or 17 or 18 or 20 or 21 or 22 or 23 or 24 or 25 or 26 or 27 or 28  30. omnivor*.mp.  31. (conventional adj3 diet*).mp.  32. (normal adj3 diet*).mp.  33. (regular adj3 diet*).mp.  34. (mixed adj3 diet*).mp.  35. exp Meat/  36. exp Eggs/  37. exp Egg Proteins, Dietary/  38. exp Dairy Products/  39. exp Milk/  40. exp Milk Proteins/  41. (meat* adj1 protein*).mp.  42. (meat* adj1 product*).mp.  43. (animal* adj1 protein*).mp.  44. (animal* adj1 product*).mp.  45. (fish* adj1 protein*).mp.  46. (fish* adj1 product*).mp.  47. (poultry adj1 protein*).mp.  48. (poultry adj1 product*).mp.  49. (chicken* adj1 protein*).mp.  50. (chicken* adj1 product*).mp.  51. (egg* adj1 protein*).mp.  52. (egg* adj1 product*).mp.  53. (milk adj1 protein*).mp.  54. (milk adj1 product*).mp.  55. (dairy adj1 protein*).mp.  56. (dairy adj1 product*).mp.  57. 30 or 31 or 32 or 33 or 34 or 35 or 36 or 37 or 38 or 39 or 40 or 41 or 42 or 43 or 44 or 45 or 46 or 47 or 48 or 49 or 50 or 51 or 52 or 53 or 54 or 55 or 56  58. OGTT.mp.  59. 'oral glucose tolerance test'.mp.  60. exp Hemoglobin A, Glycosylated/  61. hba1c.mp.  62. fructosamine*.mp.  63. insulin*.mp.  64. glycemia.mp.  65. exp Glucose/  66. exp Hyperglycemia/  67. hyperinsulin*.mp.  68. dysglycemia.mp.  69. gly* albumin.mp.  70. exp diabetes mellitus/  71. metabolic syndrome.mp.  72. HOMA*.mp.  73. 58 or 59 or 60 or 61 or 62 or 63 or 64 or 65 or 66 or 67 or 68 or 69 or 70 or 71 or 72  74. 29 and 57 and 73  75. 74 not (exp infant formula/ or exp milk, human/) |

* For all databases the original search was 19 December 2013; updated searches were performed
10 November 2014 and 26 August 2015.

**Table S2.** Study quality assessment using the Heyland Methodological Quality Score (MQS) *.

| **Study, Year (Reference)** | **Design ^†^** | | | **Sample ^‡^** | | | **Intervention ^§^** | | | **MQS (*n*/13)** |
| --- | --- | --- | --- | --- | --- | --- | --- | --- | --- | --- |
|  | **Randomization (*n*/2)** | **Blinding (*n*/1)** | **Analysis (*n*/2)** | **Selection (*n*/1)** | **Compatibility (*n*/1)** | **Follow-up (*n*/1)** | **Protocol (*n*/1)** | **Co-interventions (*n*/2)** | **Crossovers (*n*/2)** |  |
| TYPE 2 DIABETES |  |  |  |  |  |  |  |  |  |  |
| Anderson *et al.*, 1998 [26] | 1 | 0 | 2 | 0 | 1 | 1 | 1 | 2 | 0 | 8 |
| Hermansen *et al.*, 2001 [16] | 1 | 1 | 0 | 0 | 1 | 0 | 1 | 1 | 0 | 5 |
| Wheeler *et al.*, 2002 [15] | 2 | 0 | 0 | 0 | 1 | 0 | 1 | 2 | 0 | 6 |
| Azadbakht *et al.*, 2008 [13] | 1 | 0 | 0 | 0 | 1 | 0 | 1 | 1 | 0 | 4 |
| Azadbakht *et al.*, 2009 [28] | 1 | 0 | 0 | 0 | 1 | 0 | 1 | 1 | 0 | 4 |
| Gobert *et al.*, 2010 [29] | 2 | 1 | 0 | 1 | 1 | 0 | 1 | 1 | 0 | 7 |
| Cohen et al, 2011 [27] | 1 | 0 | 2 | 0 | 1 | 1 | 0 | 2 | 0 | 7 |
| Miraghajani *et al.*, 2013 [21] | 2 | 0 | 0 | 0 | 1 | 0 | 1 | 2 | 0 | 6 |
| Abd-Mishani *et al.*, 2014 [30] | 1 | 0 | 0 | 0 | 1 | 0 | 0 | 2 | 0 | 4 |
| Hosseinpour-Niazi *et al.*, 2014 [14] | 2 | 0 | 0 | 0 | 1 | 0 | 1 | 2 | 0 | 6 |
| Markova *et al.*, 2015 [20] ^\|\|^ | - | - | - | - | - | - | - | - | - | - |
| TYPE 1 DIABETES |  |  |  |  |  |  |  |  |  |  |
| Kontessis *et al.*, 1995 [24] | 1 | 0 | 2 | 0 | 1 | 1 | 0 | 2 | 0 | 7 |
| Stephenson *et al.*, 2005 [25] | 1 | 0 | 0 | 1 | 1 | 0 | 1 | 1 | 0 | 5 |

MQS = Heyland Methodological Quality Score; * The Heyland MQS assigns a score of 0 or 1 or from 0 to 2 over 9 categories of quality related to study design, sampling procedures, and interventions, for a total of 13 points. Trials that scored ≥8 were considered to be of higher quality [19]; ^†^ Randomization was scored 2 points for being randomized with the methods described, 1 point for being randomized without the methods described, or 0 points for being neither randomized nor having the methods described. Blinding was scored 1 point for being double-blind or 0 points for “other.” Analysis was scored 2 points for being intention-to-treat; all other types of analyses scored 0 points; ^‡^ Sample selection was scored 1 point for being consecutive eligible or 0 points for being preselected or indeterminate. Sample comparability was scored 1 point for being comparable or 0 points for not being comparable at baseline. Follow-up was scored 1 point for being 100% or 0 points for <100%; ^§^ Treatment protocol was scored 1 point for being reproducibly described or 0 points for being poorly described. Co-interventions were scored 2 points for being described and equal, 1 point for being described but unequal or indeterminate, or 0 points for not being described. Treatment crossovers (where participants were switched from the control treatment to the experimental treatment) were scored 2 points for being <10%, 1 point for being >10%, and 0 points for not being described; ^||^ Study quality was not assessed for this study since data was limited (the study’s conference abstract and correspondence with the authors were the only sources of available data).

**Table S3.** Continuous *a priori* & post-hoc subgroup analyses for HbA_1c_.

| **Subgroup Category** | **Range** | **No. of Trials ^†^** | **N** | **β (95% CI) ^‡^** | **Residual *I*^2^ ^§^** | ***p*-Value** |
| --- | --- | --- | --- | --- | --- | --- |
| Baseline HbA_1c_ | 5.9–8.5% | 8 | 133 | −0.04 (−0.27, 0.19) | 0.00% | 0.695 |
| Percent AP replaced | 4–70 %/day | 8 | 157 | 0.00 (−0.01, 0.01) | 0.00% | 0.877 |
| Grams AP replaced | 2.4–64 g/day | 8 | 133 | 0.00 (−0.00, 0.01) | 0.00% | 0.294 |
| Absolute fiber intake ^\|\|^ | 12.6–41 g/day | 6 | 112 | 0.00 (−0.02, 0.02) | 0.00% | 0.751 |
| Between arm ∆ in fiber intake ^¶^ | −1.4–4.9 g/day | 6 | 112 | −0.04 (−0.23, 0.15) | 0.00% | 0.611 |
| Within arm ∆ in fiber intake ^#^ | −1.8–15 g/day | 3 | 61 | −0.00 (−0.52, 0.52) | 56.60% | 0.995 |
| Absolute SF intake ^\|\|^ | 7.6–10 %E | 4 | 95 | 0.01 (−0.26, 0.27) | 14.48% | 0.907 |
| Between arm ∆ in SF intake ^¶^ | −3.6–0 %E | 4 | 95 | 0.15 (−0.42, 0.72) | 0.00% | 0.381 |
| Within arm ∆ in SF intake ^#^ | −3.28 – −0.23 %E | 2 | 41 | - | - | - |
| Follow-up | 4–12 weeks | 9 | 170 | −0.02 (−0.08, 0.03) | 0.00% | 0.346 |
| Diabetes duration ** | 3–15 years | 4 | 78 | −0.03 (−0.16, 0.11) | 0.00% | 0.467 |
| Isoflavone intake | 88–165 mg/day | 2 | 49 | - | - | - |
| Body weight | 79–111 kg | 8 | 161 | 0.00 (−0.02, 0.02) | 0.00% | 0.984 |
| Sex | 0–77.8 %W | 9 | 170 | −0.00 (−0.01, 0.01) | 0.00% | 0.549 |

AP = animal protein; E = energy; N = number of subjects; No. = number; SF = saturated fat; W = women; * Statistically significant subgroup (*p* < 0.05); ^†^ Number of trials from total number of trials (=9); ^‡^ β is the slope derived from subgroup analyses on meta-regression analyses and represents the treatment effect of replacing animal protein with plant protein for each subgroup; ^§^ The residual *I*^2^ value indicates heterogeneity unexplained by the subgroup; ^||^ Absolute intakes represent intakes within the treatment arm; ^¶^ Between arm differences represent the difference between the treatment (T) and control (C) arm (T-C); ^#^ Within arm differences represent the difference between end (E) and baseline (B) values within the treatment arm (E-B); ** Data from the trials Anderson *et al.* [26], Cohen *et al.* [27] and Kontessis *et al.* [24] were not included in this subgroup analysis since not enough information was provided to determine the mean diabetes duration.

**Table S4.** Continuous *a priori* & post-hoc subgroup analyses for fasting glucose.

| **Subgroup Category** | **Range** | **No. of Trials ^†^** | **N** | **β (95% CI)** ^‡^ | **Residual *I*^2^ ^§^** | ***p*-Value** |
| --- | --- | --- | --- | --- | --- | --- |
| Baseline fasting glucose | 6.7–10.4 mmol/L | 9 | 209 | −0.35 (−0.80, −0.10) | 24.18% | 0.109 |
| Percent AP replaced | 4–70 %/day | 9 | 205 | −0.01 (−0.03, 0.01) | 56.00% | 0.187 |
| Grams AP replaced | 6–50 g/day | 10 | 218 | −0.01 (−0.03, 0.02) | 68.19% | 0.502 |
| Absolute fiber intake ^\|\|^ | 12.6–41 g/day | 8 | 181 | 0.03 (−0.04, 0.10) | 68.38% | 0.361 |
| Between arm ∆ in fiber ^¶^ intake | −7–6 g/day | 8 | 181 | 0.04 (−0.14, 0.22) | 80.26% | 0.576 |
| Within arm ∆ in fiber intake ^#^ | −1.8–15 g/day | 5 | 116 | 0.04 (−0.29, 0.37) | 71.20% | 0.729 |
| Absolute SF intake ^\|\|^ | 2.6–9.7 %E | 5 | 127 | −0.02 (−0.80, 0.77) | 65.75% | 0.953 |
| Between arm ∆ in SF intake ^¶^ | −3.6 – −0.6 %E | 5 | 127 | 1.00 (−0.32, 2.33) | 28.52% | 0.095 |
| Within arm ∆ in SF intake ^#^ | −3.3 – −0.2 %E | 4 | 96 | 0.97 (−1.96, 3.89) | 69.72% | 0.291 |
| Follow-up | 4–208 weeks | 10 | 218 | 0.00 (−0.01, 0.00) | 84.19% | 0.241 |
| Diabetes duration ** | 3–15 years | 6 | 137 | −0.16 (−0.40, 0.08) | 31.82% | 0.122 |
| Isoflavone intake | 56–165 mg/day | 4 | 104 | 0.00 (−0.47, 0.05) | 68.87% | 0.868 |
| Body weight | 70.6–102.3 kg | 8 | 178 | 0.01 (−0.07, 0.09) | 47.63% | 0.756 |
| Sex | 28.6–77.8 %W | 10 | 218 | −0.01 (−0.04, 0.01) | 69.77% | 0.244 |

AP = animal protein; E = energy; N = number of subjects; No. = number; SF = saturated fat;
W = women; * Statistically significant subgroup (*p* < 0.05); ^†^ Number of trials from total number of trials (=10); ^‡^ β is the slope derived from subgroup analyses on meta-regression analyses and represents the treatment effect of replacing animal protein with plant protein for each subgroup; ^§^ The residual *I*^2^ value indicates heterogeneity unexplained by the subgroup; ^||^ Absolute intakes represent intakes within the treatment arm; ^¶^ Between arm differences represent the difference between the treatment (T) and control I arm (T-C); ^#^ Within arm differences represent the difference between end I and baseline (B) values within the treatment arm (E-B); ** Data from the trials Cohen *et al.* [27] and Kontessis *et al.* [24] were not included in this subgroup analysis since not enough information was provided to determine the mean diabetes duration.

**Table S5.** Continuous *a priori* & post-hoc subgroup analyses for fasting insulin.

| **Subgroup Category** | **Range** | **No. of Trials ^†^** | **N** | **β (95% CI) ^‡^** | **Residual *I*^2^ ^§^** | ***p*-Value** |
| --- | --- | --- | --- | --- | --- | --- |
| Baseline fasting insulin | 56.3–134.2 pmol/L | 5 | 118 | 0.18 (−0.65, 1.02) | 6.36% | 0.536 |
| Percent AP replaced | 4–34 %/day | 4 | 105 | 0.22 (−2.34, 2.78) | 36.54% | 0.744 |
| Grams AP replaced | 2.5–50 g/day | 5 | 118 | 0.31 (−0.45, 1.08) | 0.00% | 0.284 |
| Absolute fiber intake ^\|\|^ | 19.2–41 g/day | 4 | 105 | 0.62 (−2.68, 3.91) | 31.50% | 0.506 |
| Between arm ∆ in fiber intake ^¶^ | −1.2–4.5 g/day | 4 | 105 | −1.37 (−12.13, 9.39) | 28.58% | 0.638 |
| Within arm ∆ in fiber intake ^#^ | −1.8–15 g/day | 2 | 49 | - | - | - |
| Absolute SF intake ^\|\|^ | 7.1–9.7 %E | 2 | 60 | - | - | - |
| Between arm ∆ in SF intake ^¶^ | −1.3 – −0.6%E | 2 | 60 | - | - | - |
| Within arm ∆ in SF intake ^#^ | −0.23 %E | 1 | 29 | - | - | - |
| Follow-up | 4–12 weeks | 5 | 118 | −6.59 (−18.25, 5.06) | 0.00% | 0.170 |
| Diabetes duration ** | 3–3.4 years | 2 | 49 | - | - | - |
| Isoflavone intake | 88–165 mg/day | 2 | 49 | - | - | - |
| Body weight | 76.3–96.1 kg | 4 | 87 | 0.44 (−7.30, 8.18) | 0.00% | 0.830 |
| Sex | 30–77.4 %W | 5 | 118 | −0.30 (−0.92, 0.33) | 0.00% | 0.226 |

AP = animal protein; E = energy; N = number of subjects; No. = number; SF = saturated fat;
W = women; * Statistically significant subgroup (*p* < 0.05); ^†^ Number of trials from total number of trials (=5); ^‡^ β is the slope derived from subgroup analyses on meta-regression analyses and represents the treatment effect of replacing animal protein with plant protein for each subgroup; ^§^ The residual I^2^ value indicates heterogeneity unexplained by the subgroup; ^||^ Absolute intakes represent intakes within the treatment arm; ^¶^ Between arm differences represent the difference between the treatment (T) and control (C) arm (T-C); ^#^ Within arm differences represent the difference between end (E) and baseline (B) values within the treatment arm (E-B); ** Data from the trial Cohen *et al.* [27] was not included in this subgroup analysis since not enough information was provided to determine the mean diabetes duration.

**Table S6.** Post-hoc piecewise linear meta-regression analyses for the continuous subgroup looking at percent animal protein replaced with plant protein from total protein on fasting glucose.

| **Dose threshold,**  **% AP replaced with PP from total protein** | **Dose ranges,**  **% AP replaced with PP from total protein** | **β (95% CIs) *** | **Residual *I*^2^** † | ***p*-value** |
| --- | --- | --- | --- | --- |
| 20 | ≤20 | 0.00 (–0.13, 0.13) | 61.29% | 0.821 |
|  | >20 | –0.01 (–0.04, 0.02) |  |  |
| 25 | ≤25 | 0.01 (–0.09, 0.11) | 54.23% | 0.637 |
|  | >25 | –0.02 (–0.05, 0.02) |  |  |
| 30 | ≤30 | 0.00 (–0.06, 0.08) | 49.03% | 0.516 |
|  | >30 | –0.02 (–0.05, 0.01) |  |  |
| 35 | ≤35 | 0.00 (–0.05, 0.06) | 47.76% | 0.504 |
|  | >35 | –0.02 (–0.06, 0.01) |  |  |
| 40 | ≤40 | –0.00 (–0.05, 0.05) | 49.08% | 0.580 |
|  | >40 | –0.02 (–0.07, 0.02) |  |  |

AP = animal protein; PP = plant protein; * β is the slope derived from the piecewise linear meta-regression analyses and represents the treatment effect on fasting glucose for doses above and below each dose-threshold representing percent animal protein replaced with plant protein from total protein; † The residual *I*^2^ value indicates heterogeneity unexplained by each dose-threshold.

2. Supplementary Figures

**Figure S1.** Risk of bias assessment using Cochrane Risk of Bias Tool*. * Risk of bias=low if study design was likely to have little influence over the true outcome; unclear if insufficient information was given to assess risk; high if study design was likely to have an influential effect on the true outcome; ^†^ Random Sequence Generation assessed whether the method of randomization was described; ^‡^ Allocation concealment assessed whether investigators could tell which treatment participants were going to be randomized to; ^§^ Blinding of participants and personnel assessed whether the study was blinded to investigators, study personnel/outcome assessors, and/or participants; ^||^ Incomplete outcome data assessed whether missing outcome data effected the true outcome; ^¶^ Selected outcome reporting assessed whether all of the studies pre-specified outcomes (primary and secondary) of interest have been reported in a pre-specified way.

**Figure S2. Categorical** a priori subgroup **analyses for HbA_1c_.** AP = animal protein; *N* = number of participants; MQS = Heyland Methodological Quality Score; SF = saturated fat; * Statistically significant between subgroups (*p* < 0.05); ^†^ Point estimates for each subgroup level (diamonds) are the pooled effect estimates. The dashed line represents the pooled estimate for the overall (total) analysis; ^‡^ The residual I^2^ value indicates heterogeneity unexplained by the subgroup; ^§^ Pairwise
between-subgroup mean differences (95%CIs) for plant protein type were as follows:
0.12 (−0.43, 0.68) (1 *vs.* 2); 0.19 (−0.47, 0.84) (1 *vs.* 3); 0.00 (−0.70, 0.70) (1 *vs.* 4); −0.06 (−0.69, 0.56)
(2 *vs.* 3); 0.12 (−0.43, 0.68) (2 *vs.* 4); −0.19 (−0.95, 0.57) (3 *vs.* 4); ^||^ Absolute intakes represent intakes within the treatment arm. Between arm differences represent the difference between the treatment (T) and control (C) arm (T–C); ^¶^ Between arm differences represent the difference between the treatment (T) and control (C) arm (T-C); ^#^ Within arm differences represent the difference between end (E) and baseline (B) values within the treatment arm (E–B); ****** Pairwise between-subgroup mean differences (95%CIs) for food form were as follows: 0.13 (−0.28, 0.54) (1 *vs.* 2); 0.02 (−0.56, 0.59)
(1 *vs.* 3); 0.11 (0.40, 0.63) (2 *vs.* 3); ^††^ Diabetes complications consists of 1 or more of the following conditions: nephropathy, retinopathy, glomerular hyperfiltration.

**Figure S3.** Categorical risk of bias subgroup analyses for HbA_1c_. N = number of participants;
ROB = risk of bias; * Statistically significant between subgroups (*p* < 0.05); ^†^ Point estimates for each subgroup level (diamonds) are the pooled effect estimates. The dashed line represents the pooled estimate for the overall (total) analysis; ^‡^ The residual *I*^2^ value indicates heterogeneity unexplained by the subgroup; ^§^ Pairwise between-subgroup mean differences (95%CIs) for sequence generation were as follows: 0.23 (−0.12, 0.58) (2 *vs.* 1); −0.58 (−1.99, 0.83) (3 *vs.* 1); 0.81 (−0.58, 2.19) (2 *vs.* 3);
^||^ Pairwise between-subgroup mean differences (95%CIs) for blinding of participants, personnel, and outcome assessors were as follows: 0.12 (−0.32, 0.57) (2 *vs.* 1); −0.14 (−1.20, 0.92) (3 *vs.* 1);
0.27 (−0.83, −1.36) (2 *vs.* 3).

**Figure S4.** **Categorical** a priori subgroup **analyses for fasting glucose.** AP = animal protein;
N = number of participants; MQS = Heyland Methodological Quality Score; SF = saturated fat;
* Statistically significant between subgroups (*p* < 0.05); ^†^ Point estimates for each subgroup level (diamonds) are the pooled effect estimates. The dashed line represents the pooled estimate for the overall (total) analysis; ^‡^ The residual I^2^ value indicates heterogeneity unexplained by the subgroup; ^§^ Pairwise between-subgroup mean differences (95%CIs) for plant protein type were as follows: −0.69 (−1.88, 0.50) (1 *vs.* 2); −0.50 (−2.39, 1.39) (1 *vs.* 3); −0.56 (−1.76, 0.64) (1 *vs.* 4); −0.19 (−2.02, 1.63)
(3 *vs.* 2); −0.13 (−1.23, 0.96) (4 *vs.* 2); 0.06 (−1.77, 1.89) (4 *vs.* 3); ^||^ Pairwise between-subgroup mean differences (95%CIs) for animal protein type were as follows: 0.94 (0.46, 1.41) (2 *vs.* 1);
0.53 (0.18, 0.87) (3 *vs.* 1); 0.41 (−0.15, 0.97) (2 *vs.* 3); ^¶^ Absolute intakes represent intakes within the treatment arm; ^#^ Between arm differences represent the difference between the treatment (T) and control (C) arm (T–C); ****** Within arm differences represent the difference between end (E) and baseline (B) values within the treatment arm (E–B); ^††^ Diabetes complications consists of 1 or more of the following conditions: nephropathy, retinopathy, glomerular hyperfiltration.

**Figure S5.** Categorical risk of bias subgroup analyses for fasting glucose. *N* = number of participants; ROB = risk of bias; * Statistically significant between subgroups (*p* < 0.05); ^†^ Point estimates for each subgroup level (diamonds) are the pooled effect estimates. The dashed line represents the pooled estimate for the overall (total) analysis; ^‡^ The residual I^2^ value indicates heterogeneity unexplained by the subgroup; ^§^ Pairwise between-subgroup mean differences (95%CIs) for sequence generation were as follows: 0.48 (−0.26, 1.23) (2 *vs.* 1); −2.84 (−6.70, 1.01) (3 *vs.* 1); 3.32 (−0.54, 7.19) (2 *vs.* 3); ^||^ Pairwise between-subgroup mean differences (95%CIs) for blinding of participants, personnel, and outcome assessors were as follows: 0.28 (−0.72, 1.27) (2 *vs.* 1); 0.51 (−1.39, 2.41) (3 *vs.* 1); −0.23 (−2.13, 1.68) (2 *vs.* 3); ^¶^ Pairwise between-subgroup mean differences (95%CIs) for incomplete outcome data were as follows: 0.11 (−1.43, 1.65) (1 *vs.* 2); 0.45 (−1.23, 2.13) (1 *vs.* 3); −0.34 (−1.50, 0.82) (3 *vs.* 2); ^#^ Pairwise between-subgroup mean differences (95%CIs) for selective outcome reporting data were as follows: 0.74 (0.06, 1.43) (1 *vs.* 2); 0.62 (−0.40, 1.64) (1 *vs.* 3); 0.12 (−0.90, 1.14) (2 *vs.* 3).

**Figure S6. Categorical** a priori subgroup **analyses for fasting insulin.** AP = animal protein; *N* = number of participants; MQS = Heyland Methodological Quality Score; SF = saturated fat;
* Statistically significant between subgroups (*p* < 0.05); ^†^ Point estimates for each subgroup level (diamonds) are the pooled effect estimates. The dashed line represents the pooled estimate for the overall (total) analysis; ^‡^ The residual I^2^ value indicates heterogeneity unexplained by the subgroup; ^§^ Pairwise between-subgroup mean differences (95%CIs) for plant protein type were as follows: −26.15 (−268.74, 216.45) (3 *vs.* 2); −10.87 (−56.82, 35.08) (4 *vs.* 2); 15.28 (−227.65, 258.21) (4 *vs.* 3);
^||^ Absolute intakes represent intakes within the treatment arm; ^¶^ Between arm differences represent the difference between the treatment (T) and control (C) arm (T–C); ^#^ Within arm differences represent the difference between end (E) and baseline (B) values within the treatment arm (E–B);
****** Diabetes complications consists of 1 or more of the following conditions: nephropathy, retinopathy, glomerular hyperfiltration.

**Figure S7.** Categorical risk of bias subgroup analyses for fasting insulin. *N* = number of participants; ROB = risk of bias; * Statistically significant between subgroups (*p* < 0.05); ^†^ Point estimates for each subgroup level (diamonds) are the pooled effect estimates. The dashed line represents the pooled estimate for the overall (total) analysis; ^‡^ The residual I^2^ value indicates heterogeneity unexplained by the subgroup; ^§^ Pairwise between-subgroup mean differences (95%CIs) for blinding of participants, personnel, and outcome assessors were as follows: −20.35 (−274.86, 234.16) (1 *vs.* 2); −31.67 (−302.72, 239.38) (1 *vs.* 3); 11.32 (−88.11, 110.75) (3 *vs.* 2).

**Figure S8.** Funnel plot for trim-and-fill analysis of HbA_1c._ The horizontal line represents the pooled effect estimate expressed as a mean difference, the diagonal lines represent the pseudo 95% CIs of the mean difference and the clear circles represent effect estimates for each included study.

**Figure S9.** Funnel plot for trim-and-fill analysis of fasting glucose. The horizontal line represents the pooled effect estimate expressed as a mean difference, the diagonal lines represent the pseudo−95% CIs of the mean difference and the clear circles represent effect estimates for each included study while back squares represent "imputed" studies.

**Figure S10.** Funnel plot for trim-and-fill analysis of fasting insulin. The horizontal line represents the pooled effect estimate expressed as a mean difference, the diagonal lines represent the pseudo−95% CIs of the mean difference and the clear circles represent effect estimates for each included study.
